# Three Prospective Case Studies Examining Mifepristone's Efficacy in Patients with Treatment-Resistant PTSD

**DOI:** 10.1155/2024/4768647

**Published:** 2024-04-26

**Authors:** Agnes van Minnen, Lizelotte Vos, Pierre M. Bet, Ad de Jongh, Felix Linsen, Hein J. F. van Marle, Onno C. Meijer, Willem M. Otte, Marije Russcher, Christiaan H. Vinkers

**Affiliations:** ^1^Psychotrauma Expertise Centre (PSYTREC) Bilthoven, Bilthoven, Netherlands; ^2^Behavioural Science Institute (BSI), Radboud University, Nijmegen, Netherlands; ^3^Department of Clinical Pharmacology and Pharmacy, Amsterdam University Medical Center, Vrije Universiteit Amsterdam, Amsterdam, Netherlands; ^4^Academic Centre for Dentistry Amsterdam (ACTA), University of Amsterdam and VU University Amsterdam, Amsterdam, Netherlands; ^5^School of Health Sciences, Salford University, Salford, Manchester, UK; ^6^Institute of Health and Society, University of Worcester, Worcester, UK; ^7^School of Psychology, Queen's University, Belfast, UK; ^8^Department of Psychiatry, Amsterdam University Medical Center, Vrije Universiteit Amsterdam, Amsterdam, Netherlands; ^9^Amsterdam, Neuroscience, Mood, Anxiety, Psychosis, Sleep and Stress Program, Amsterdam, Netherlands; ^10^ARQ National Psychotrauma Center, Diemen, Netherlands; ^11^GGZ inGeest Mental Health Care, Amsterdam, Netherlands; ^12^Department of Medicine, Division Endocrinology, Leiden University Medical Center (LUMC), Leiden, Netherlands; ^13^Department of Child Neurology, UMC Utrecht Brain Center, University Medical Center Utrecht, Utrecht, Netherlands; ^14^Department of Hospital Pharmacy, Meander Medical Center, Amersfoort, Netherlands

## Abstract

Despite the availability of various treatment approaches for patients with posttraumatic stress disorder (PTSD), some patients do not respond to these therapies, and novel treatment approaches are needed. This study investigated the efficacy of mifepristone, a glucocorticoid receptor antagonist, in treatment-resistant PTSD patients. Three patients with PTSD who were resistant to standard psychological and pharmacological treatments were prescribed mifepristone (600–1,200 mg/day) for 1 week. A baseline-controlled single-case design was used, involving a 2-week baseline phase (no intervention), a 1-week intervention phase (mifepristone), and a 2-week postintervention phase. The primary outcome measure, self-reported PTSD symptom severity (PCL-5), was assessed daily, with participants providing their own control condition. Two of the three patients experienced a significant reduction in PTSD symptom severity after the intervention phase and no longer met the diagnostic criteria for PTSD. These positive results were maintained during long-term follow-up. These findings support the potential effectiveness of mifepristone in the treatment of patients with treatment-resistant PTSD. However, our findings must be interpreted with caution, and further studies with larger sample sizes and more rigorous designs are necessary to confirm the promising results.

## 1. Introduction

Not all patients with posttraumatic stress disorder (PTSD) respond to evidence-based psychological and pharmacological treatments [1–3], and treatment resistance is common [4, 5]. Animal studies have suggested that dysregulation of glucocorticoid receptors (GR) and cortisol signaling is associated with the development and persistence of PTSD-like symptoms [6, 7]. The involvement of cortisol and GR in the stress response has led to two different lines of thought regarding the enhancement of treatment in patients with PTSD. One is cortisol administration during trauma-focused psychotherapy [8], and the second line involves “resetting” the stress-system by antagonizing the GR. The concept is that strong stressors (especially during certain sensitive periods) lead to long-lasting changes in the responsiveness of brain circuits in a GR-dependent manner. Consequently, a GR antagonist could improve PTSD outcomes by either blocking ongoing GR activation (competition with one's own hormone in occupation of the GR) or by reversal of GR-induced transcriptional processes, such as the set points of the HPA axis [9]. In support of this, in a pilot clinical study with four male veteran patients with PTSD, the GR antagonist mifepristone significantly reduced the severity of PTSD symptoms at posttreatment compared to placebo [10]. However, a recently published controlled study only found benefits with the same mifepristone treatment in veterans with PTSD without traumatic brain injury (TBI) [11]. These studies included only veterans, and the patients were not resistant to treatment. To further explore the effects of the GR antagonist mifepristone in a different PTSD population with treatment resistance, we conducted a baseline-controlled single-case study of three patients who had previously shown treatment resistance to standard PTSD therapies, as prescribed in international treatment guidelines.

## 2. Materials and Methods

### 2.1. Participants

Participants were three patients diagnosed with PTSD who showed treatment resistance, which was defined as (1) fulfilling DSM-5 diagnostic criteria for PTSD according to the Clinician-Administered PTSD Scale (CAPS-5) and (2) stage two treatment resistance as proposed by Sippel et al. [12]: “nonresponse to at least three evidence-based treatments recommended by a recent clinical practice guideline, at least one of which is a full course of trauma-focused psychotherapy.”

### 2.2. Design

**Figure 1:**
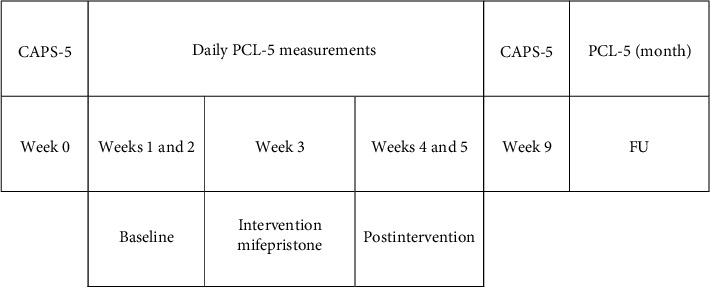
Measurement schedule. *Note*. CAPS, clinician-administered PTSD scale; PCL-5, PTSD checklist for DSM-5; FU, follow-up.

A baseline-controlled single-case design was used, consisting of a baseline phase of 2 weeks (no intervention), an intervention phase of 1 week (mifepristone tablets, 600 or 1,200 mg as indicated, administered once daily in the morning), followed by a postintervention phase of 2 weeks (see Figure 1). The primary outcome measure was measured daily in each phase for each case, and the participants provided their own control conditions. The diagnostic criteria for PTSD with CAPS-5 were also measured at baseline and postintervention.

### 2.3. Outcome Measures

The primary outcome measure was self-reported PTSD symptom severity, measured using the Dutch version of the PTSD Checklist (PCL-5) [13]. The PCL-5 has high internal consistency and good validity [14]. The secondary outcome measure was PTSD diagnosis based on the DSM-5 [15], as measured using the Dutch version of the Clinician-Administered PTSD Scale (CAPS-5-past month version [16]).

### 2.4. Statistical Analysis

Bayesian inference is well suited for inferring ongoing sequentially retrieved day-to-day *n*-of-1 trial data. We analyzed the individual PCL-5 score series of all patients using Bayesian modeling. First, we checked which distribution family best fits the data (e.g., Gaussian, Poisson, negative binomial, or multinomial). We also checked whether correction for autocorrelation (i.e., serial correlation between PCL-5 scores, which could violate the assumed independence of individual data points in standard linear regression models) was required. We found that the negative binomial distribution family best fit all three n-of-1 trials, and no correction for autocorrelation (e.g., with an additional autoregressive (AR (1) model term) was required. Bayesian linear negative binomial regression models (Stan language) were fitted with noninformative priors (“brms” package, R 4.2.1; [17]). For each patient, we tested two hypotheses: H_0_, there would be no change in the total PCL-5 score between baseline and postintervention, and H_1_, there would be a change in total PCL-5 score between baseline and postintervention. We quantified the evidence for one hypothesis over the other with the Bayes factor. A Bayes factor of >1 indicates that the data under consideration more strongly support H_1_ than H_0_, whereas a Bayes factor of <1 implies no evidence for H_1_. We characterized the difference in the PCL-5 score as the mean with a Bayesian 95% credible interval based on the posterior distribution. The CAPS-5 total scores and diagnostic states were descriptively reported and interpreted. Raw data and scripts are publicly available at https://github.com/wmotte/mifepristone-case-series.

## 3. Case 1: Tim

Tim, aged 61, was a former police officer with multiple work-related traumatic experiences, such as exposure to fire and physical violence. He experienced these traumatic experiences during his whole career as a policeman, and 4 years ago, he was diagnosed with PTSD. Due to his severe PTSD symptoms, Tim has been unable to perform his duties as a police officer for the past 4 years. In addition to the PTSD symptoms, the patient experienced severe physical pain and unexplained somatic symptoms. Prior to receiving the diagnosis of PTSD, the patient exhibited no symptoms of psychopathology. He was prescribed mifepristone 600 mg/day for 7 days.

### 3.1. Results

**Figure 2:**
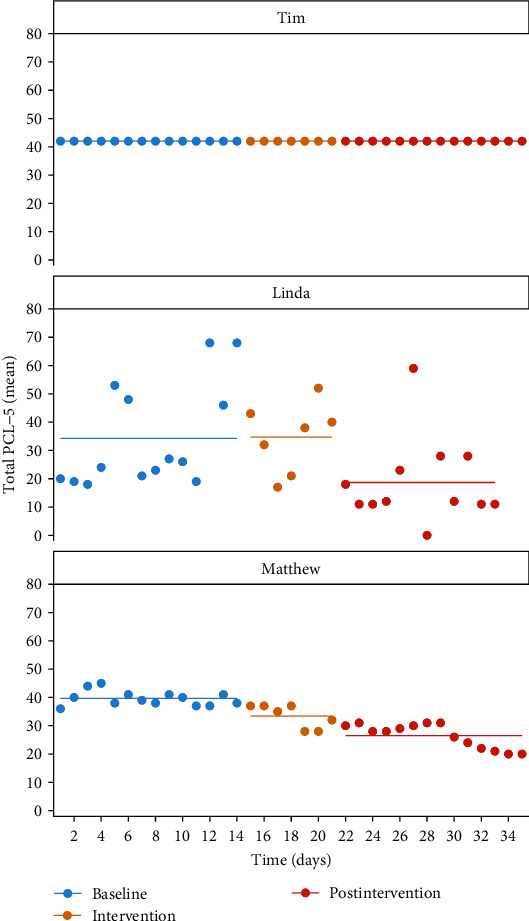
Total scores of PTSD Checklist for DSM-5 during 1 week of mifepristone treatment, preceded by 2 weeks of baseline and followed by 2 weeks postintervention for the three cases. The dots represent the scores, and the horizontal lines represent the period-based mean.

Figure 2 (top) shows the measurement results for Tim. Visual inspection of the data revealed no notable differences in PCL-5 scores between the phases. Using Bayesian analysis, a Bayes factor in favor of H_1_ of 0.16 was found for Tim, indicating that H_1_ was rejected, and no positive effects were found for mifepristone. The absence of variation in the data may suggest a tendency toward a “straight-lining response style,” potentially compromising the validity of the data [18]. At follow-up 1 year later, the total PCL score (past month) was 40. At baseline, the total CAPS-5 score was 42. Postintervention, Tim's CAPS-5 total score was 40, and he continued to meet all the diagnostic criteria for PTSD.

### 3.2. Patient Experience

Tim reported that he did not notice any significant effects during the intervention or any side effects. He was disappointed, and after the intervention, Tim indicated that nothing had changed in his PTSD symptomatology. The patient still felt significantly affected and restrained in functioning because of his PTSD symptoms.

### 3.3. Discussion

One possible explanation for Tim's nonresponse is that the dosage he received may have been too low, as a mifepristone dosage of 600 mg/day has a lower chance of reaching high plasma levels than 1,200 mg/day, and a lower plasma level was related to decreased efficacy for psychotic and depression outcomes in several clinical trials [19]. Therefore, he was offered another week of treatment with a higher dose of mifepristone (1,200 mg/day), but declined this offer.

## 4. Case 2: Linda

Linda, aged 47, worked as a paramedic, and her traumatic experiences included being confronted with severe injuries and dead bodies multiple times during her work. However, her index trauma was multiple childhood abuse, which commenced at the age of 4 by a close family member and a friend's father. Due to her severe hyperarousal symptoms, she has been unable to work for the past 6 years. Before being diagnosed with PTSD, the patient showed no signs of psychopathology. She was prescribed mifepristone at a daily dose of 600 mg for 7 days. We hypothesized that if her hyperarousal symptoms decreased, Linda would respond well to trauma-focused treatment, and we expected mifepristone to augment this effect. Therefore, we offered four sessions of trauma-focused treatment during this phase. This treatment was provided during the second week of the postintervention period, on days 28 and 29, using the period after the last dose of mifepristone as a window of opportunity for trauma-focused treatment to work. The measurement scores for Linda are presented in Figure 2 (middle).

### 4.1. Results

Visual inspection revealed a notable difference in PCL-5 scores between the baseline and postintervention phases, with lower PCL-5 total scores observed during the postintervention period. Using Bayesian analysis, a Bayes factor of 9.1 in favor of H_1_ was found for Linda, indicating support for the hypothesis that mifepristone reduced total PCL-5 scores. The average score reduction was −15.78 (95% CI: −31.66 to −1.63). However, she reported experiencing severe tiredness, nausea, postponement of menstruation, and momentary loss of control as side effects during the first few days of mifepristone use. The adverse effects disappeared over the course of the week. At the beginning of the baseline phase, Linda's CAPS-5 score was 49. Seven weeks after using mifepristone, Linda no longer met the diagnostic criteria for PTSD, and her total CAPS-5 score was 13. At follow-up 1 year later, her PCL score (past month) had remained low (total score 10).

### 4.2. Patient Experience

After a week of treatment with mifepristone, Linda reported a profound shift in her overall emotional state. She described feeling like she had been reset to default mode, characterized by a notable absence of stress and the constant need for vigilance. She experienced improved sleep and no longer felt compelled to check her children's safety frequently. One year after the treatment, Linda enjoyed a much greater sense of relaxation and well-being. She could easily perform her work duties.

### 4.3. Discussion

It is difficult to disentangle the precise effects of mifepristone and trauma-focused sessions 1 week after its use. Nevertheless, we observed a decrease in PCL-5 total scores before trauma-focused treatment began on day 28, suggesting that mifepristone was responsible for reducing PTSD symptoms rather than trauma-focused treatment sessions alone. We cannot entirely discount the possibility of placebo effects; however, given the patient's subjective experience of feeling less stressed, it appears that mifepristone may have helped her respond to the trauma-focused treatment. Moreover, the results were sustained during the follow-up.

## 5. Case 3: Matthew

Matthew, aged 63 years, has worked as a train driver for the past 30 years, during which he has witnessed several train accidents and encountered deceased individuals on multiple occasions. Three years prior, he was diagnosed with PTSD, and due to posttraumatic stress symptoms, he was unable to continue working. In light of this, he decided to take early retirement. Before receiving the diagnosis of PTSD, the patient did not exhibit any indications of psychological disorders. He received 1,200 mg of mifepristone daily for 1 week.

### 5.1. Results

Upon visual inspection, it was observed that Matthew's PCL-5 scores were somewhat lower during the week he used mifepristone and notably lower during the postintervention phase than during the baseline phase (Figure 2, bottom). The average score reduction was −13.14 (95% CI: −17.97 to −8.39). The mean PCL-5 score during the postintervention phase was below the cutoff score of 33 for PTSD diagnosis. These findings align with the Bayesian analysis, which strongly supports H_1_ with a Bayes factor >100, indicating that PCL-5 scores were significantly lower in the postintervention phase than in the baseline phase.

Matthew did not report any side effects. At the start of the baseline phase, the CAPS-5 total score was 34. Four weeks after the discontinuation of mifepristone, the patient no longer met the diagnostic criteria for PTSD according to the CAPS-5, with a total score of 16. At the 4-month follow-up, the total PCL-5-score (last month's version) was 29, slightly higher than his last score and lower than that at pretreatment.

### 5.2. Patient Experience

After 1 week of mifepristone use, Matthew reported feeling less tense, being able to sleep throughout the night, and engaging in activities again. His wife confirmed that he was less irritable and vigilant. Although he still reported some trauma-related symptoms, he reported being able to cope with them, that they no longer affected his daily life and functioning, and that he did not require further trauma-focused treatment. Matthew indicated he felt very happy with the results 4 months after using mifepristone. His wife confirmed good results at the follow-up.

### 5.3. Discussion

Matthew showed good results from the mifepristone, and similar to Linda in Case 2, reported a reduction of symptoms in the “alterations in arousal and reactivity” symptom cluster; he felt more calm, less on edge, and could finally sleep much better. Although at follow-up 4 months later, his symptoms slightly increased again, the treatment results are still noteworthy and clinically important.

## 6. General Discussion

Two of three patients reported positive outcomes from using mifepristone and even showed clinical remission according to the CAPS-5 scores. Although the treatment duration was short, only 1 week, and the symptoms reduced rapidly, these improvements lasted 4–12 months. Moreover, the reported side effects were minimal or subsided quickly. The lack of response in Case 1 (Tim) may have been due to the low dose of mifepristone. Although the optimal dose of mifepristone for the treatment of patients with PTSD is unknown, clinical trials in patients with psychosis and depression have suggested that high plasma levels of mifepristone are associated with better treatment outcomes, with 1,200 mg being the most likely dose to achieve optimal plasma levels [19].

The strengths of this study included its time-controlled design, long-term follow-up, and well-evaluated clinical measures. Moreover, while previous studies on mifepristone have primarily focused on veteran populations, our research studied the effects of mifepristone in patients with treatment-resistant PTSD who had experienced trauma distinct from that experienced by veterans. However, the absence of randomization, placebo control, and small sample size can be considered limitations of our study. Furthermore, the additional trauma-focused treatment in Case 2′s postintervention phase limited the conclusions regarding the effects of mifepristone alone, and it is unknown whether mifepristone may produce beneficial effects, particularly in combination with trauma-focused psychotherapy.

In conclusion, our three case studies confirm that modulating GR may be a promising treatment option for treatment-resistant patients with PTSD, although larger placebo-controlled studies are necessary. Clinical trials on the effects of GR antagonists in patients with PTSD are ongoing worldwide (e.g., [20]). However, it is important to note that research conducted in a specific population of veterans may have certain limitations because some veterans may have TBI, which may introduce confounding factors that can impact the observed outcomes of mifepristone and complicate the interpretation of the results. For example, a recent randomized controlled study using mifepristone in veterans showed negative results [11]. This may have been the result of the relatively low dose (600 mg), predominantly male population, and particularly, the large proportion of veterans with TBI. A significant TBI × mifepristone interaction was found, with veterans without TBI showing clinically relevant mifepristone effects at weeks 4 and 12. Therefore, researchers recommend further exploration of the effects of mifepristone in non-TBI populations. It would also be worthwhile to expand these trials to include patients with PTSD who have experienced other trauma histories, such as sexual abuse, female patients, and patients who have shown treatment resistance. Therefore, as a follow-up, we plan to conduct a placebo-controlled RCT with treatment-resistant mixed-sex patients with PTSD who are not veterans.

## Data Availability

Raw data and scripts are publicly available at https://github.com/wmotte/mifepristone-case-series.

## Additional Points

*Highlights*. A glucocorticoid receptor antagonist, mifepristone, shows promise in PTSD treatment. This medication was tested in three patients with treatment-resistant PTSD. Two of the three patients experienced a significant reduction in PTSD symptoms. The effects remained during long-term follow-up. Mifepristone caused minimal side effects and can be considered safe.
